# Ambient Air Pollution Associations with Retinal Morphology in the UK Biobank

**DOI:** 10.1167/iovs.61.5.32

**Published:** 2020-05-19

**Authors:** Sharon Y. L. Chua, Anthony P. Khawaja, Andrew D. Dick, James Morgan, Baljean Dhillon, Andrew J. Lotery, Nicholas G. Strouthidis, Charles Reisman, Tunde Peto, Peng T. Khaw, Paul J. Foster, Praveen J. Patel, on behalf of The UK Biobank Eye and Vision Consortium

**Affiliations:** ^1^ National Institute for Health Research, Biomedical Research Centre at Moorfields Eye Hospital National Health Service Foundation Trust & University College London Institute of Ophthalmology, London, UK; ^2^ University College London, Institute of Ophthalmology, London, UK; ^3^ Bristol Medical School Translational Health Sciences, University of Bristol, Bristol, UK; ^4^ School of Optometry & Vision Sciences, Cardiff University, Cardiff, Wales, UK; ^5^ Centre for Clinical Brain Sciences, School of Clinical Sciences, University of Edinburgh, Edinburgh, UK; ^6^ National Health Service Lothian Princess Alexandra Eye Pavilion, Edinburgh, UK; ^7^ Clinical and Experimental Sciences, Faculty of Medicine, University of Southampton, Southampton, UK; ^8^ Topcon Healthcare Solutions Research & Development, Oakland, New Jersey, United States; ^9^ School of Medicine, Dentistry and Biomedical Sciences, Queens University Belfast, Belfast, UK

**Keywords:** retinal layers, OCT, air pollution

## Abstract

**Purpose:**

Because air pollution has been linked to glaucoma and AMD, we characterized the relationship between pollution and retinal structure.

**Methods:**

We examined data from 51,710 UK Biobank participants aged 40 to 69 years old. Ambient air pollution measures included particulates and nitrogen oxides. SD-OCT imaging measured seven retinal layers: retinal nerve fiber layer, ganglion cell–inner plexiform layer, inner nuclear layer, outer plexiform layer + outer nuclear layer, photoreceptor inner segments, photoreceptor outer segments, and RPE. Multivariable regression was used to evaluate associations between pollutants (per interquartile range increase) and retinal thickness, adjusting for age, sex, race, Townsend deprivation index, body mass index, smoking status, and refractive error.

**Results:**

Participants exposed to greater particulate matter with an aerodynamic diameter of <2.5 µm (PM_2.5_) and higher nitrogen oxides were more likely to have thicker retinal nerve fiber layer (β = 0.28 µm; 95% CI, 0.22–0.34; *P* = 3.3 × 10^−20^ and β = 0.09 µm; 95% CI, 0.04–0.14; *P* = 2.4 × 10^−4^, respectively), and thinner ganglion cell–inner plexiform layer, inner nuclear layer, and outer plexiform layer + outer nuclear layer thicknesses (*P* < 0.001). Participants resident in areas of higher levels of PM_2.5_ absorbance were more likely to have thinner retinal nerve fiber layer, inner nuclear layer, and outer plexiform layer + outer nuclear layers (β = –0.16 [95% CI, –0.22 to –0.10; *P* = 5.7 × 10^−8^]; β = –0.09 [95% CI, –0.12 to –0.06; *P* = 2.2 × 10^−12^]; and β = –0.12 [95% CI, –0.19 to –0.05; *P* = 8.3 × 10^−4^], respectively).

**Conclusions:**

Greater exposure to PM_2.5_, PM_2.5_ absorbance, and nitrogen oxides were all associated with apparently adverse retinal structural features.

Air pollution is a global and major public health problem: the global Burden of Diseases, Injuries, and Risk factors Study reported that air pollution accounts for 6.7 million deaths globally in 2016.^1^ Air pollutants are a complex mixture of small solid or liquid particles of varying composition in the atmosphere. Previous studies provide compelling evidence of increased mortality and morbidity with exposure to higher concentrations of air pollutants.^1^^,^^2^ Exposure to air pollution is associated with respiratory disease, cardiovascular disease,^3^ neurologic diseases,^4^ and eye diseases, including glaucoma^5^^,^^6^ and AMD.^7^ The potential mechanisms of air pollution-induced health effects include oxidative stress, activation of inflammatory pathways, and increased coagulation.^8^^–^^10^ The retina is susceptible to oxidative stress owing to its high consumption of oxygen, high proportion of polyunsaturated fatty acids, and its exposure to visible light.^11^ Additionally, oxidative damage increases with age, resulting in retinal dysfunction and cell loss. Therefore, the ageing retina is potentially particularly susceptible to damage from air pollution.

SD-OCT is a noninvasive imaging technique that allows visualization of the multilayered architecture of the retina, and measurement of individual retinal sublayers including the retinal nerve fiber layer (RNFL), ganglion cell–inner plexiform layer (GCIPL), photoreceptor layer, and RPE.^12^ Changes in the thickness measurements of retinal layers are important because they provide useful information for detecting and diagnosing retinal diseases such as AMD, glaucoma, and diabetic retinopathy.^13^^–^^17^ Understanding the impact of air pollution on the retinal structures may provide insights into age-related eye diseases.

We examined data from UK Biobank, a large community-based cohort study. The aim of our study was to evaluate the relationship of ambient air pollution at participants’ residential address with retinal layer thicknesses at the macula as measured using SD-OCT.

## Methods

### Study Population

UK Biobank is a very large community-based cohort of 502,656 UK residents registered with the National Health Service and aged 40 to 69 years at enrolment. Baseline examinations were carried out between 2006 and 2010 at 22 study assessment centers. The North West Multi-center Research Ethics Committee approved the study in accordance with the principles of the Declaration of Helsinki. The overall study protocol (www.ukbiobank.ac.uk/resources/) and protocols for individual tests (http://biobank.ctsu.ox.ac.uk/crystal/docs.cgi) are available online.

### Participant Characteristics

Participants answered a wide-ranging touch-screen questionnaire covering demographic, socioeconomic, lifestyle, and systemic and ocular diseases information. The choices for race/ethnicity include white (English/Irish or other white background), Asian or British Asian (Indian/Pakistani/Bangladeshi or other Asian background), black or black British (Caribbean, African, or other black background), Chinese, mixed (white and black Caribbean or African, white and Asian, or other mixed background), or other ethnic group (not defined). Race/ethnicity was classified into two categories (white vs. non-white). The Townsend deprivation index was determined according to the participants’ postcodes at recruitment and the corresponding output areas from the preceding national census. The index was calculated based on the output area's employment status, home and car ownership, and household condition; the higher and more positive the index, the more deprived an area. Smoking status was classified into three categories (never, previous, and current). Physical measures included height and weight. Body mass index (BMI) was defined as weight divided by height squared.

### Ocular Assessment

Ocular assessment was introduced as an enhancement in 2009 for the six assessment centers, which are spread across the UK.^18^ The refractive error of both eyes was measured by an autorefractor (Tomey RC 5000, Nagoya, Japan).^19^ Spherical equivalent refraction (SER) was calculated as sphere power plus half cylinder power. High-resolution SD-OCT imaging was performed using the Topcon 3D OCT 1000 Mk2 (Topcon Inc, Oakland, NJ) in a dark room, without pupillary dilation. This system has an axial resolution of 6µm and OCT images were obtained using a raster scan protocol, 6 mm × 6 mm in area, centered on the fovea. This raster scan consisted of 128 B-scans, each consisted of 512 A-scans. The inner and outer retinal surfaces were segmented using the Topcon Advanced Boundary Segmentation Algorithm (Version 1.6.1.1).^20^^,^^21^ Quality control measures during data collection included (1) image quality score, (2) internal limiting membrane indicator, (3) validity count, and (4) motion indicators. These quality control measured have been described previously and have been incorporated to international consensus reporting guidelines on OCT metrics.^22^^–^^24^ In brief, the image quality score indicates the signal strength for the scan. The internal limiting membrane indicator identifies blinks and scans that contain regions of severe signal attenuation or segmentation errors. The validity count indicator identifies scans with significant degree of clipping in the scan's *z*-axis dimension. The motion indicator identifies blinks, eye motion artefacts, and segmentation failures.

**Figure 1.**
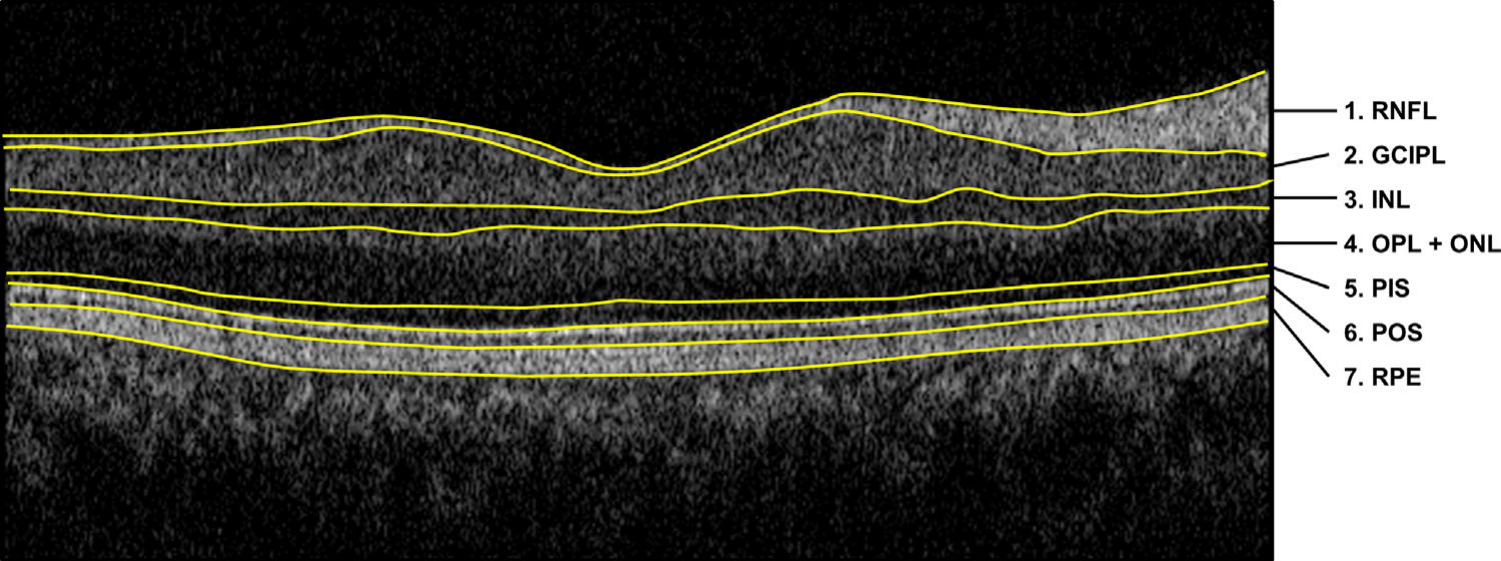
OCT images acquired using Topcon 3D OCT-1000. The seven retinal layers are as follows: (1) RNFL, (2) GCIPL, (3) INL, (4) OPL+ONL, (5) PIS, (6) POS, and (7) RPE.

The Topcon Advanced Boundary Segmentation algorithm was used to segment and generate the average thickness of the seven retinal layers on the SD-OCT. Seven retinal layers were identified by the automatic segmentation algorithm: RNFL (layer 1), GCIPL (layer 2), inner nuclear layer (INL) (layer 3), outer plexiform layer + outer nuclear layer (OPL+ONL) (layer 4), photoreceptor inner segment (PIS) (layer 5), photoreceptor outer segment (POS) (layer 6), and RPE (layer 7) (Fig. 1).^22^

### Air Pollution Measurements

The air pollution measures were provided by the Small Area Health Statistics Unit (www.sahsu.org/) as part of the BioSHaRE-EU Environmental Determinants of Health Project (www.bioshare.eu/), and were linked centrally to the assessment data by UK Biobank analysts (http://biobank.ctsu.ox.ac.uk/crystal/docs/EnviroExposEst.pdf). Detailed measures of air pollution parameters have been published elsewhere .^25^^,^^26^ The annual average concentration of PM_2.5_ (aerodynamic diameter of <2.5 µm), PM_2.5__–__10_ (aerodynamic diameter between 2.5 and 10 µm, PM_10_ (aerodynamic diameter of <10 µm), PM_2.5_ absorbance ([PM_2.5_ ab] a measurement of the blackness of PM_2.5_ filter – a proxy for elemental or black carbon), nitrogen dioxide (NO_2_), and nitrogen oxides (NO_x_) were calculated centrally by the UK Biobank using a land use regression model developed by the European Study of Cohorts for Air Pollution Effects (ESCAPE) project (www.escapeproject.eu/).^27^ By using the predictor variables obtained from the Geographic Information System such as traffic, land use, and topography, the land use regression models calculate the spatial variation of annual average air pollution concentration at participants’ residential addresses given at baseline visit. NO_2_ annual concentration data was available for 4 years (2005, 2006, 2007, and 2010), and PM_10_ data were available for 2007 and 2010. We averaged the values to obtain the mean estimate. All other particulate matter (PM) and nitrogen pollutants had the exposure data for a single year (2010).

### Inclusion and Exclusion Criteria

Participants were excluded from the analysis based on the following criteria: (1) participants who withdrew consent; or (2) had self-reported diabetes-related eye disease, eye injury resulting in vision loss or other serious eye conditions; high SER (<–6 diopters [D] or >+6D); or (3) participants who had poor SD-OCT signal strength, image quality score of less than 45, poor centration certainty, or poor segmentation certainty using Topcon Advanced Boundary Segmentation software.^22^^,^^24^ These participants were excluded because of the well-recognized impact these conditions have on retinal layer thickness.^28^

### Statistical Analysis

For this analysis, if both eyes of a patient were eligible for inclusion, one eye was randomly selected using STATA software (version 13, StataCorp LP, College Station, TX). We compared the baseline characteristics of participants included in the study to those excluded from the study, using χ^2^ or *t*-test as appropriate for the variable. The mean and standard deviation of the individual retinal layers were calculated. Multivariable linear regression analyses were performed to determine the associations between each air pollutant (independent variables) and individual retinal thickness (dependent variables), adjusting for age, sex, race, Townsend deprivation index, BMI, smoking status, and SER. The effect estimates represent the change in retinal layers variables per interquartile range increment in air pollution. We conducted a test for interaction, to evaluate the effect of PM × NO_x_ on the retinal layers. Additionally, in sensitivity analyses, we analyzed the associations of each air pollutant with the GCL and IPL. In view of our broad hypothesis that six pollutant classes may potentially influence any of seven retinal sublayer thicknesses, statistical significance was set at *P* < 0.001 after Bonferroni correction (*P* = 0.05/[6 × 7]).

## Results

**Figure 2.**
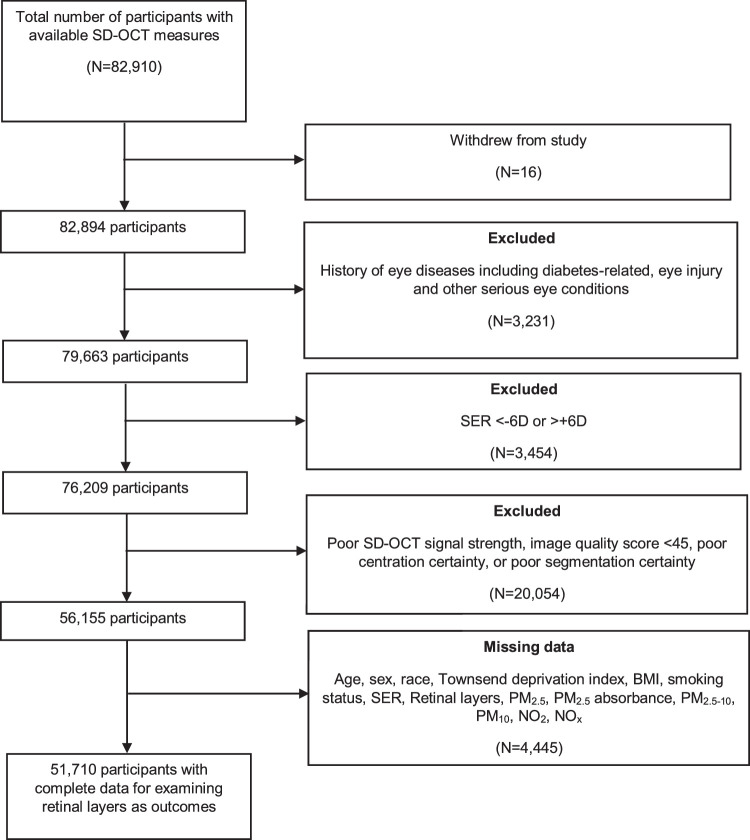
Flowchart of participants included in the study.

Of the 82,894 participants with available data on retinal layers, 26,739 participants were excluded according to the excluded criteria, and 4445 participants were further excluded owing to missing data (age, sex, race, Townsend deprivation index, BMI, smoking status, SER or any of the individual retinal layers) (Fig. 2). Hence, 51,710 participants were included in the analysis.

Table 1 shows a comparison of participants included compared with those excluded from the study. Given the very large sample size, even small differences between the groups were statistically significant. Compared with participants excluded from the study, those included were slightly younger, more likely to be male and white, had more negative Townsend deprivation index (implies less degree of deprivation), had a lower BMI, were more likely to be smokers, and had more positive SER. The distribution of ambient air pollution exposure for each outcome-specific group is shown in Table 2. The median concentrations are higher for NO_x_ and NO_2_ than for PM. The mean of the different retinal layers are: RNFL, 29.13 ± 5.26 µm; GCIPL, 73.22 ± 6.34 µm; INL, 32.57 ± 2.38 µm; OPL+ONL, 80.44 ± 6.39 µm; PIS, 23.79 ± 1.91 µm; POS, 38.01 ± 4.22 µm; and RPE, 25.32 ± 3.76 µm.

**Table 1.** Comparison of Characteristics Between Participants Included and Excluded From the Study

Characteristics	Included (*n* = 51,710)	Excluded (*n* = 31,184)	*P* Value
Sociodemographic factors			
Age	56.4 ± 8.1	57.4 ± 7.9	<0.001
Sex			
Men	24,367 (47.1)	13,979 (44.8)	
Women	27,343 (52.9)	17,205 (55.2)	<0.001
Race			
White	47,660 (92.2)	28,055 (91.5)	
Non-white	4050 (7.8)	2608 (8.5)	0.001
Townsend deprivation index	−1.20 ± 2.9	−1.16 ± 3.0	0.043
Clinical factors			
BMI (kg/m^2^)	27.2 ± 4.4	27.3 ± 4.5	0.009
Smoking status			
Never	28,744 (55.6)	17,525 (57.1)	
Previous	18,107 (35.0)	10,520 (34.2)	
Current	4859 (9.4)	2664 (8.7)	<0.001
Spherical equivalent (D)	−0.002 ± 2.0	−0.90 ± 3.6	<0.001

Numbers are mean ± SD or number (%), unless otherwise stated.

Student *t*-test or χ^2^ test where appropriate (2-sided).

Among those excluded, there were missing race data for 521 participants, missing Townsend deprivation index data for 90 participants, missing BMI data for 1168 participants, missing smoking status data for 475 participants, and missing spherical equivalent data for 619 participants.

**Table 2.** Distribution of PM_2.5,_ PM_2.5_ ab_,_ PM_2.5__–__10,_ PM_10,_ NO_2_, and NO_X_ Among Participants Included in the Study

Air Pollutants (µg/m^3^)	Median (IQR)	Range
PM_2.5_	9.92 (1.12)	8.17–19.69
PM_2.5_ ab	1.25 (0.33)	0.83–3.71
PM_2.5__–__10_	6.46 (0.76)	5.57–11.30
PM_10_	19.72 (2.78)	13.38–29.30
NO_2_	32.38 (12.64)	9.44–86.65
NO_X_	44.72 (14.97)	19.74–263.96

IQR, interquartile range.

Table 3 shows the multivariable regression analysis between ambient air pollution and inner retinal layers. Higher concentrations of PM_2.5_ and NO_x_ were associated with a thicker RNFL and a thinner GCIPL (all *P* < 0.001). By contrast, higher levels of PM_2.5_ ab and PM_10_ were associated with a thinner RNFL, and higher exposure to PM_10_ was associated with a thicker GCIPL (all *P* < 0.001). Higher concentrations of PM_2.5_, PM_2.5_ ab, PM_10_, NO_2_, and NO_x_ were associated with a thinner INL (*P* < 0.001).

**Table 3.** Multivariate Linear Regression of Ambient Air Pollution With Thickness of the Inner Retinal Layers

	RNFL			GCIPL			INL		
Air Pollutants (µg/m^3^)	β (µm)	95% CI	*P* Value	β (µm)	95% CI	*P* Value	β (µm)	95% CI	*P* Value
PM_2.5_	0.28	(0.22 to 0.34)	**3.3 × 10^−^^20^**	−0.51	(−0.59 to −0.44)	**6.3 × 10^−^^47^**	−0.07	(−0.10 to −0.04)	**1.1 × 10^−^^7^**
PM_2.5_ absorbance	−0.16	(−0.22 to −0.10)	**5.7 × 10^−^^8^**	0.05	(−0.01 to 0.12)	0.12	−0.09	(−0.12 to −0.06)	**2.2 × 10^−^^12^**
PM_2.5-10_	0.006	(−0.03 to 0.05)	0.78	0.05	(0.005 to 0.10)	0.030	−0.01	(−0.03 to 0.002)	0.09
PM_10_	−0.28	(−0.35 to −0.22)	**7.4 × 10^−^^17^**	0.38	(0.30 to 0.46)	**1.2 × 10^−^^21^**	−0.13	(−0.15 to −0.10)	**2.8 × 10^−^^17^**
NO_2_	−0.07	(−0.14 to 0.0005)	0.050	0.04	(−0.05 to 0.12)	0.38	−0.14	(−0.17 to −0.11)	**5.9 × 10^−^^19^**
NO_X_	0.09	(0.04 to 0.14)	**2.4 × 10^−^^4^**	−0.21	(−0.27 to −0.16)	**5.2 × 10^−^^13^**	−0.05	(−0.07 to −0.03)	**2.8 × 10^−^^6^**

The beta coefficients represent per interquartile range increase in exposure variable.

Adjusted for age, sex, race, Townsend deprivation index, BMI, smoking status and refractive error.

Bold values denote statistical significance at the p<0.001 level.

Table 4 shows the multivariable regression analysis between ambient air pollution and outer retinal layers. Higher concentrations of PM_2.5_, PM_2.5_ ab, and NO_x_ were associated with a thinner OPL+ONL thickness (*P* < 0.001). By contrast, higher concentrations of PM_2.5_ ab and NO_2_ were associated with thicker POS thickness. None of the air pollutants were significantly associated with PIS and RPE thickness after accounting for multiple testing. Table 5 shows a summary of the association of higher ambient air pollution concentration with thickness of the seven retinal layers. Of all the air pollutants, PM_2.5_ had the strongest association with RNFL, GCIPL, and OPL+ONL thickness, although PM_10_ also showed similar effect as PM_2.5_ on RNFL. NO_2_ had the strongest association with INL and POS thickness. In sensitivity analyses, PM_2.5_ had the strongest effect on GCL and IPL, higher levels of PM_2.5_ were associated with a thinner GCL and IPL (β = –0.39 µm, *P* = 2.8 × 10^−57^ and β = –0.13 µm, *P* = 2.0 × 10^−18^, respectively). Similarly, higher levels of NO_x_ were associated with a thinner GCL and IPL (*P* < 0.001). Exposure to higher concentrations of PM_2.5_ ab and PM_10_ were associated with a thicker GCL (*P* < 0.001). Our data indicate there was an interaction effect between PM_2.5_ and NO_x_ with RNFL and GCIPL (*P* = 1.1 × 10^−21^ and *P* = 1.9 × 10^−15^, respectively).

**Table 4.** Multivariate Linear Regression of Ambient Air Pollution With Thickness of the Outer Retinal Layers

	OPL + ONL			PIS			POS			RPE		
Air pollutants (µg/m^3^)	β (µm)	95% CI	*P* Value	β (µm)	95% CI	*P* Value	β (µm)	95% CI	*P* Value	β (µm)	95% CI	*P* Value
PM_2.5_	−0.18	(−0.25 to −0.11)	**7.8 × 10^−^^7^**	0.03	(0.006 to 0.05)	0.014	0.06	(0.01 to 0.11)	0.013	−0.08	(−0.12 to −0.03)	0.001
PM_2.5_ ab	−0.12	(−0.19 to −0.05)	**8.3 × 10^−^^4^**	0.03	(0.01 to 0.05)	0.003	0.11	(0.06 to 0.16)	**4.9 × 10^−^^6^**	−0.05	(−0.10 to −0.008)	0.022
PM_2.5–10_	−0.03	(−0.08 to 0.02)	0.27	−0.005	(−0.02 to 0.01)	0.49	−0.02	(−0.05 to 0.01)	0.29	−0.02	(−0.05 to 0.01)	0.25
PM_10_	−0.07	(−0.15 to 0.007)	0.07	−0.01	(−0.03 to 0.01)	0.43	0.08	(0.02 to 0.13)	0.005	−0.04	(−0.09 to 0.02)	0.16
NO_2_	−0.08	(−0.17 to −0.001)	0.047	0.04	(0.02 to 0.07)	0.001	0.16	(0.10 to 0.22)	**2.0 × 10^−^^8^**	−0.05	(−0.11 to 0.0001)	0.05
NO_X_	−0.11	(−0.16 to −0.05)	**4.3 × 10^−^^4^**	0.02	(0.004 to 0.04)	0.017	0.05	(0.009 to 0.09)	0.017	−0.02	(−0.06 to 0.02)	0.36

The beta coefficients represent per interquartile range increase in exposure variable.

Adjusted for age, sex, race, Townsend deprivation index, BMI, smoking status and refractive error.

Bold values denote statistical significance at the p<0.001 level.

**Table 5.** The Effect of an Increase in Ambient Air Pollution on the Thickness of Each Retinal Layer

	Inner Retinal Layers			Outer Retinal Layers			
Air Pollutants (µg/m^3^)	RNFL	GCIPL	INL	OPL+ONL	PIS	POS	RPE
PM_2.5_	**↑↑**	**↓↓**	**↓**	**↓↓**	NS	NS	NS
PM_2.5_ ab	**↓**	NS	**↓**	**↓**	NS	**↑**	NS
PM_2.5__–__10_	NS	NS	NS	NS	NS	NS	NS
PM_10_	**↓↓**	**↑**	**↓**	NS	NS	NS	NS
NO_2_	NS	NS	**↓↓**	NS	NS	**↑↑**	NS
NO_X_	**↑**	**↓**	**↓**	**↓**	NS	NS	NS

The arrows represent either an increase (**↑)** or decrease (**↓)** in the thickness of each retinal layer after an increase in exposure to air pollution and indicates a significant association. Double arrows indicate the specific air pollutant has the strongest effect on the individual retinal layer and shows a significant association. For example, of all the air pollutants on GCIPL, the effect of PM_2.5_ is the strongest on GCIPL. A nonsignificant association between the air pollutant and retinal layer will be indicated as nonsignificant (NS).

Statistical significance was set at *P* < 0.001 after Bonferroni correction.

## Discussion

In this large study of UK Biobank participants, we have identified associations between ambient outdoor air pollutant levels at participants’ residential addresses and concurrent measures of individual retinal layers. We identified the following: (1) higher levels of PM_2.5_, PM_2.5_ ab, PM_10_, and NO_x_ were associated with potentially adverse features in inner retinal layer thicknesses (except between PM_2.5_ ab and GCIPL); (2) higher levels of PM_2.5_, PM_2.5_ ab, NO_2_, and NO_x_ were associated with either or both thinner OPL+ONL and thicker POS; (3) PM_2.5__–__10_ was not associated with retinal layer measures; and (4) there was no association between ambient air pollution and PIS and RPE after there was no association between ambient air pollution and PIS and RPE after Bonferroni correction. To our knowledge, this is the first study to report the association of ambient air pollution with individual retinal structures.

Most of the health effects of air pollution are mediated by inhalation.^2^ The deposition of PM in the respiratory tract is mainly influenced by the aerodynamic particle size. The smaller the particle, the greater its ability to penetrate the respiratory system, enter the bloodstream, cross the blood–brain barrier, and access the central nervous system.^29^ Because the retina is a part of the central nervous system, PM may cause neuroglial damage and inflammatory responses in the retinal structures. Neurologic diseases such as Parkinson's disease and Alzheimer's disease, as well as ocular disease, including glaucoma,^30^ are characterized by a loss of retinal ganglion cells, the only retinal neurons that project to the brain through the optic nerve.^31^^,^^32^ Our data indicate that higher levels of PM_2.5_ and NO_x_ were particularly associated with thicker RNFL and thinner GCIPL. It is relevant that metallic and organic toxins, which are likely to covary with pollution indices, have been shown to decrease axoplasmic transport in the optic nerve.^33^^,^^34^ It is possible that moderate, sustained toxic effects compromise axoplasmic flow in the RNFL, resulting in the observed greater thickness in this layer. Consistent with this finding, it is notable that diesel exhaust particulates administered by intratracheal instillation (200 mg/L) in rats induced a significant and acute increase in the thickness of the inner plexiform, inner and outer nuclear, and rod/cone cell layers. In the same study, RNFL and GCLs showed no difference in thickness, although capillary congestion was noted.^35^ We have previously reported that elevated IOP is associated with a thinner GCIPL in UK Biobank participants notably with no association between the level of IOP or RNFL thickness.^36^ A thinner retinal GCL has been documented in ethambutol toxicity and tobacco/alcohol toxicity, compared with measures from healthy controls.^37^ A thinner GCIPL may be due to oxidative stress causing cell death.^38^

NO_x_ contributes to the formation of ground-level ozone. There is evidence that ozone may potentiate the adverse effects of diesel exhaust particulates, suggesting a potential interaction between PM and NO_x_^39^ and a plausible explanation for the similar effect trend for both PM_2.5_ and NO_x_. Our results indicate there was an interaction effect between PM_2.5_ and NO_x_ with the RNFL and the GCIPL. Conversely, higher concentrations of PM_2.5_ ab and PM_10_ were associated with a thinner RNFL, whereas higher levels of PM_10_ were associated with a thicker GCIPL. Although thinner RNFL measurements may be related to loss of retinal ganglion cell axons from longer term, lethal injury, the bidirectional effects observed in the RNFL are difficult to explain. Retinal ganglion cells death induced by reactive oxygen species can occur through several mechanisms, such as protein modification and DNA damage. Alternatively, exposure to ultrafine ambient nanoparticles in the environment may cause indirect DNA damage by signaling through gap junction proteins after generation of mitochondrial free radicals.^40^ Because approximately 50% of the RGCs are located in the macula and their cells bodies are 10 to 20 times their axons in diameter,^41^ the greater effect of ambient air pollution on the GCIPL relative to the RNFL is to be expected. RGCs dendrites in the IPL form synapses with the bipolar, amacrine, and Müller glial cells located in the INL.

Neurons in INL layer are involved in retinal homeostasis. Müller cells in particular respond to retinal damage by changing their morphology,^42^^,^^43^ which is consistent with the association between a thinner INL and higher ambient air pollution. Oxidative stress increases RPE lipofuscin,^44^ the main component of drusen. Additionally, photoreceptor synaptic terminals and photoreceptor nuclei are often decreased in regions overlying drusen,^45^ which may explain the associations between higher levels of PM_2.5_, PM_2.5_ ab, and NO_x_ with thinner OPL, ONL, and RPE thicknesses. However, it is important to note that the effect estimates were not statistically significant with RPE after Bonferroni correction under our “hypothesis-free” approach. Because the RPE cells are responsible for daily phagocytosis of POS, RPE dysfunction may increase POS thickness. This finding may provide a plausible explanation for the greater POS thickness observed with greater exposure to PM_2.5_ ab and NO_2_. Our data indicate that greater ambient PM_2.5_ exposure was the most strongly associated with three retinal layers (Table 5). By contrast, PM_2.5__–__10_ had no discernible effect on any retinal layers. The adverse health effects observed by fine PM (PM_2.5_) compared with coarse PM (PM_2.5__–__10_) may be explained by the absorption of fine PM into the bloodstream through alveolar capillaries causing systemic inflammation.^10^^,^^46^ Fine PM mainly result from combustion processes^47^ and combustion-related particles are known to be more toxic to health, causing airway and systemic inflammation^48^ and myocardial ischemia,^49^ compared with particles not generated by combustion.^50^ Although, the effect size of the associations between air pollution and individual retinal layers is small and is a fraction of the axial pixel-resolution of the Topcon SD-OCT (axial resolution of 6 µm), we believe that our findings are less likely to be due to measurement error for the following reasons. First, we have only included participants with high-quality OCT images by applying strict quality control criteria, thus improving OCT segmentation accuracy. Second, the large sample size of our study allowed us to obtain greater precision of our effect estimates.

We have previously reported an association between air pollution and glaucoma.^6^ However, the present study reveals broader associations between atmospheric pollution and apparently adverse retinal structure. It is not clear whether pollution is driving the primary pathologic processes in this common disease. Even so, it is reasonable to consider that the pollutants studied here may have an additive or synergistic effect on the pathophysiology of this eye disease.

The strengths of this study include its large sample size and the high resolution and reproducibility of SD-OCT measurements of retinal thickness. The study is the first large-scale attempt to evaluate the association of ambient air pollution with inner and outer retinal structures. The UK Biobank has limitations in that it is based on a volunteer cohort, and participants are likely to be healthier and belong to a higher socioeconomic group than the general population. Although our results may not, therefore, be entirely representative of the UK population, the exposure–disease relationships are still valid.^51^ Because air pollution measures were collected before OCT data, it is most likely a nondifferential misclassification bias and will therefore skew the associations toward the null. Finally, analysis was based on a 51,170 of 82,894 participants with OCT data, which increases the risk of selection bias. However, the baseline characteristics (Table 1) of included and excluded participants were similar, with the exception of participants with a high SER (<–6 D or >+6D) who were excluded from the analysis.

In conclusion, our findings suggest that inner retinal layers may be affected by air pollution compared with outer retinal layers. Fine PM seem to have more adverse effects on the retinal structures, which may predispose to the development of common eye diseases such as macular degeneration and glaucoma. Our findings demonstrate morphologic evidence that may precede the potential damaging effects of ambient air pollution on eye disease,^5^^–^^7^ even with relatively low levels of exposure. Further studies are required to assess the relative contributions of outdoor and indoor ambient air pollution measures on retinal structure and function.
